# Sorafenib in a patient with advanced hepatocellular carcinoma and serious impairment of left ventricular function: a case report

**DOI:** 10.1186/1757-1626-2-9133

**Published:** 2009-12-02

**Authors:** Chiara Valsuani, Olimpia Siclari, Andrea Camerini, Maria Laura Canale, Marianna Rondini, Sara Donati, Paolo Puccinelli, Gianna Tartarelli, Cheti Puccetti, Domenico Amoroso

**Affiliations:** 1 Medical Oncology Division, Versilia Hospital, via Aurelia 335, 55041 Lido di Camaiore (LU), Italy; 2 Cardiology Division, Ospedale San Sebastiano, via Circondaria 1, 42015 Correggio (RE), Italy

## Abstract

**Introduction:**

sorafenib, a tyrosine-kinase inhibitor, is widely used in the treatment of advanced hepatocellular carcinoma. Drug-related toxicities are generally mild but sorafenib, as other similar agents, may induce elevation of systemic arterial blood pressure levels in relation to an interaction with cardiovascular system probably mediated by HIF pathway. This side effect may be particularly critical for patients with underlying serious heart disease as it can induce acute heart failure, a life-threatening condition, and usually such patients are excluded from active treatment with tyrosine-kinase inhibitors. We report the case of a patient affected by advanced hepatocellular carcinoma and serious impairment of cardiac function treated with sorafenib without any worsening of heart function. To our knowledge this is the first report of this kind in the literature.

**Case presentation:**

We report the case of a 74-year-old patient affected by advanced multifocal HCV-cirrhosis related hepatocellular carcinoma and severe post-ischemic fall of left-ventricular function with serious risk of cardiac functional impairment. The patient presented with an ECOG performance status of 0. Blood chemistry tests showed a substantial elevation of α-fetoprotein values and slight increases of bilirubin, of γ-GT and of GOT; the absence of encephalopathy and ascites and the normality of coagulation parameters and of albumin led to classify the patient into the functional class Child-Pugh A. The patients was successfully treated with sorafenib at the reduced daily dose of 400 mg for long-time without any worsening of heart function.

**Conclusion:**

The presented case can offer to oncologists a clinical support to take into consideration when deciding to treat with sorafenib advanced hepatocellular carcinoma patients presenting with serious impairment of cardiac function that are usually excluded from an active treatment.

## Introduction

Hepatocellular carcinoma (HCC) is ranked the fifth tumor type worldwide, with an incidence of about 620,000 new cases per year[1]. Notwithstanding most cases are detected in Asia and Africa, in highly developed countries of the Western area its incidence is continuously growing[1]. Until the very recent past, the treatment of advanced HCC with conventional antineoplastic cytotoxic drugs never resulted in significant outcomes[2,3]. However, subsequent progresses achieved in the understanding and in the elucidation of the bio-molecular mechanisms underlying the growth of HCC prompted the development of new targeted agents, thus disclosing promising opportunities for the treatment of this highly vascularized tumor in which the inhibition of angiogenesis is likely to represent the main potential therapeutic target. In this regard, sorafenib (Nexavar^®^, Bayer), an oral multikinase inhibitor targeting several tyrosine-kinase receptors involved in both blood vessel development and tumor growth[4,5], is the first and the only drug which has proved to be able to induce a statistically significant prolongation of the overall survival in patients with advanced HCC[6]. Sorafenib has shown a good safety profile, being the most common side-effects mainly represented by mild or moderate asthenia, diarrhea, skin rash with or without desquamation, and hand-foot skin reactions[6]. However, sorafenib can also interfere with the cardiovascular system inducing elevation of blood pressure values, and, in a pivotal randomized trial, cardiac events such as ischemia or myocardial infarction limited in severity to grades 2 or 3 have been observed in about 3% of patients[6]. Consequently, this evidence must be taken into serious consideration when facing a treatment with sorafenib in patients displaying cardiovascular comorbidities[7,8].

Here below we report a case of a patient with multifocal HCC HCV-cirrhosis related who, notwithstanding the presence of a series of cardiovascular alterations, has been successfully treated with sorafenib without any further significant worsening of the cardiovascular function.

## Case presentation

In May 2008, a 74-year-old heavily smoker Caucasian man was referred to our institution following ultrasonography showing multiple lesions ranging in diameter from 0.3 to 2.0 cm in both liver lobes, without any evidence of ascites. The patient was work retired in good state of health (ECOG PS 0), with an history of HCV-related liver cirrhosis well balanced while familiar history was not medically relevant. He reported a previous history of cardiovascular illness consisting in chronic ischemic post-infarction cardiomyopathy leading to a substantial decrease in left-ventricular ejection fraction (LVEF), arterial hypertension, obesity (98 kg weight and 174 cm height), stroke with cerebral vascular disease and alterations of respiratory system (chronic obstructive pulmonary disease).

**Figure 1:**
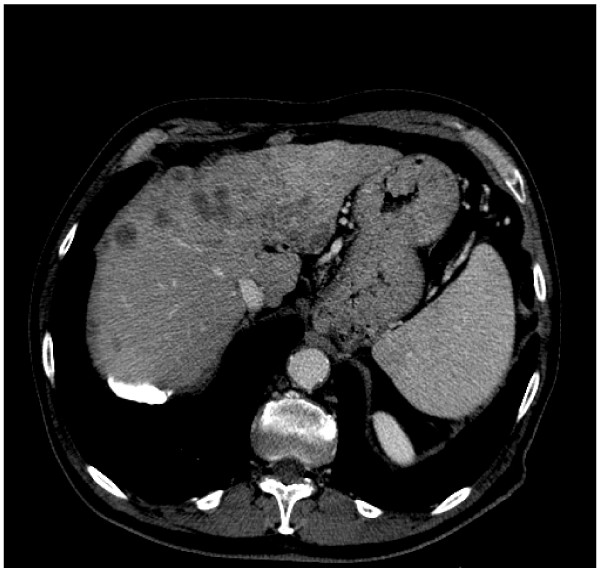
**CT-scan of upper abdomen showing multiple hepatic lesions**. Pre-treatment hepatic evaluation performed at the time of initial diagnosis by CT-scan showed multiple hepatic lesion ranging from 0.3 to 2.0 cm in both liver lobes

Total-body CT scan confirmed the presence of the hepatic lesions previously observed at ultrasonography without any other evidence deserving consideration (Figure 1). Blood chemistry tests showed a substantial elevation of α-fetoprotein values (1500 mg/ml), and slight increases of bilirubin (1.21 mg/ml), of γ-GT (116 U/L) and of GOT (76 U/L); the absence of encephalopathy and ascites, and the normality of coagulation parameters and of albumin led to classify the patient into the functional class Child-Pugh A. The echo-guided liver biopsy and the subsequent histo-pathological assessment definitely confirmed the diagnosis of HCC.

At the objective clinical examination the patient only displayed slight indolent hepatomegaly. He also showed a good hemodynamic steadiness: no signs of heart failure such as declivous edema, jugular turgor and pulmonary noises have been observed. In addition, no important symptoms, particularly those related to dyspnea or workload angina were complained of. The instrumental cardiac function assessment confirmed the serious systolic dysfunction with a LVEF of 35%. The patient was on treatment with bisoprolol, furosemide, ramipril and anti-aldosterone drugs.

Based on these evidences, the disease was categorized as stage C according to the Barcelona-Clinic Liver Cancer (BCLC) classification and, consequently, only amenable to the best available systemic treatment; therefore, starting June 2008, a treatment with sorafenib continuous dosing at the reduced dose of 400 mg/day was started.

**Figure 2:**
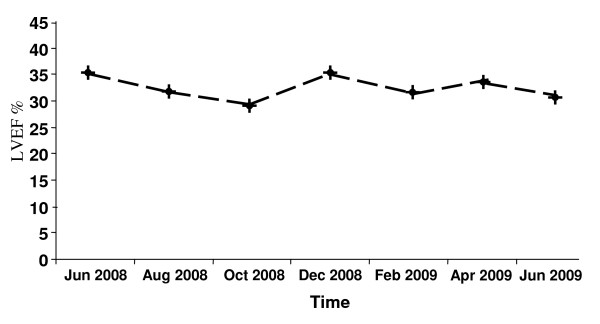
**Behaviour of left ventricular ejection fraction values during treatment**. left ventricular ejection fraction, echocardiographically evaluated every two months for one year during treatment with sorafenib, showed no further drops or significant fluctuations from baseline value. Similarly, the patient remained on good hemodynamic steadiness all treatment long.

Instrumental assessments with ultrasound carried out every two months showed a substantial unchanging of the size of the hepatic lesions. Conversely, α-fetoprotein values constantly and progressively decreased up to 260 ng/ml in June 2009, while the other hematochemical parameters showed an improvement of liver function indexes. Presently the patient is continuing the treatment with sorafenib at the same dose. Treatment was extremely well tolerated without, with the exception of asthenia, the onset of any peculiar drug-related side-effect requiring medical interventions, drug-dose reductions or treatment discontinuation. Due to the particular patient's cardiac conditions, the echographic evaluation of LVEF was carried out every two months: as it appears in Figure 2, no further drops or significant fluctuations have been observed, and the values of LVEF substantially remained stable over the time.

## Discussion

The results of the pivotal phase III study comparing sorafenib versus placebo in patients with advanced HCC (SHARP study)[6] have unquestionably modified the natural history of this disease which, on account of its high chemo-resistance, until recently was considered incurable. This new tyrosine kinase inhibitor, in fact, has been able to induce a statistically significant improvement of patients' overall survival with a 31% reduction of risk of death. Besides the undeniable efficacy data, the SHARP study also highlighted the very good safety profile of sorafenib which induced an acceptable rate of drug-related adverse events mainly consisting of constitutional symptoms, dermatologic and gastrointestinal events mostly mild to moderate in severity: and these findings are in line with the incidence of sorafenib-associated adverse events observed in studies carried out in other tumor types such as renal cell carcinoma[7,8]. However, it must be borne in mind that the use of tyrosine kinase inhibitors could be also associated with a risk of a series of cardiac events, whose patho-physiological underlying mechanism still needs to be clarified and understood. An hypothesis to explain this peculiarity is based on the role played by hypoxia-inducible factor α (HIFα) as response mediator of myocardium to acute or chronic ischemia, of myocardial re-modelling, of post-infarction re-vascularization and of vascular permeability: an HIFα reduction induced by tyrosine kinase inhibitors could interfere with these pathways thus inducing an impairment of the normal physiology of cardiovascular system [9-11].

As far as sorafenib is concerned, a survey of data on 2276 patients treated in phase I, II and III studies indicates that the incidence of congestive heart failures, the worse cardiac situation, globally accounts for 1.89%, and this figure decreases to 1.36% when only extremely serious situations are considered[12]. In another investigation carried out in an unselected population with advanced renal cell carcinoma treated with two different tyrosine kinase inhibitors, sorafenib and sunitinib[12], the reported incidence of treatment-related LVEF reductions has been 21,4% for sorafenib and 54.5% for sunitinib. Compared to the data coming from clinical trials, these rather high incidences could be explained by the particular baseline characteristics of patients showing cardiovascular clinical signs in 33.8% of cases, and by a pre-existing ventricular dysfunction in 12%. LVEF reductions, however, were temporary and regressed following treatment discontinuation[13]. Recently, a prospective phase I study to evaluate the cardiovascular safety profile of sorafenib was carried out in 53 patients with different solid tumors or lymphomas treated at the standard dose of 400 mg bid: neither significant decreases of LVEF evaluated by MUGA scan nor significant alterations of heart rate or of QTc interval have been observed[14].

Referring to our case report, at baseline the patient displayed substantial cardiovascular disorders along with a significant LVEF reduction classified NYHA II. Owing to the reduced cardiac function and to the lack of more reliable safety data concerning patients with HCC treated with sorafenib, a softer treatment consisting in a daily total dose of 400 mg was planned. After 12 months of administration, this regimen did not induce any noteworthy alteration of cardiac function and the LVEF is still classified NYAH II. In this regard it must be outlined that the median duration of treatment in both randomized studies carried out in HCC[6] and in renal cancer[7] was 5.5 months. Therefore, our possibility to administer sorafenib for 8 months without any discontinuation further strengthen the safety profile of the drug. All these evidences strongly indicate that even in high risk patients due to cardiovascular complications, such as the impairment of LVEF, a treatment with sorafenib is feasible and safe provided that a close and careful monitoring of parameters of cardiac function is done.

## Consent

Written informed consent was obtained from the patient for publication of this case report and accompanying images. A copy of the written consent is available for review by the Editor-in-Chief of this journal.

## Competing interests

The authors declare that they have no competing interests.

## Authors' contributions

CV patient data collection and manuscript writing.

OS patient data collection.

AC bibliographic research and manuscript writing.

MLC bibliographic research, echographic evaluations and cardiological data collection.

MR patient data collection.

SD patient data collection.

PP patient data collection.

GT bibliographic research.

CP bibliographic research.

DApatient data collection and manuscript writing.

All authors read and approved the final manuscript.
